# Visit to general practitioners as a proxy for accessing chronic benefits by members of medical schemes, South Africa

**DOI:** 10.4102/phcfm.v4i1.419

**Published:** 2012-10-08

**Authors:** Mncedisi M. Willie, Monwabisi Gantsho

**Affiliations:** 1 Council for Medical Schemes, South Africa

## Abstract

**Background:**

Prescribed Minimum Benefits is a list of conditions that all medical schemes need to cover in full, and includes a select of chronic conditions. Chronic conditions affect people's lifestyles and require ongoing management over a period of years for long-term survival.

**Objectives:**

This study examined the association between prevalence of selected chronic diseases and health service use, in particular visits to general practitioners (GPs) by medical scheme members.

**Method:**

This was a retrospective study on medical schemes data. The median imputation method was employed to deal with missing and unreported chronic diseases prevalence. Multivariate logistic regression analysis was employed to assess effects of chronic disease prevalence, age stratum and scheme size on GP visits per annum.

**Results:**

The study showed that prevalence of asthma was significantly associated with more than three GP visits (OR = 1.081; 95% CI = 1.008–1.159), as was prevalence of type 2 diabetes (OR = 1.087; 95% CI = 1.027–1.152), whilst prevalence of hyperlipidaemia (OR = 0.92; 95% CI = 0.875–0.97) was more likely to be associated with less than three GP visits. Prevalence of hypertension was associated with more than three GP visits per year (OR = 1.132; 95% CI = 1.017–1.26).

**Conclusion:**

This study shows that scheme size, prevalence of chronic diseases such as asthma, type 2 diabetes, hyperlipidaemia and hypertension are related to GP visits. GPs and managed care programmes employed by schemes should give special attention to certain disease states with high prevalence rates in an effort to better manage them.

## Abstrait

**Les visites chez les médecins généralistes comme indicateur de l'accès à la prise en charge des maladies chroniques des membres des régimes d'assurance maladie, Afrique du Sud**

**Contexte:**

Les prestations minimums prescrites sont une liste de maladies que tous les régimes d'assurance maladie doivent totalement prendre en charge, notamment une sélection de maladies chroniques. Les maladies chroniques affectent le mode de vie des personnes et nécessitent un suivi permanent pendant des années pour une survie à long terme.

**Objectifs:**

Cette étude examine l'association entre la prévalence des maladies chroniques et l'utilisation des services de santé, en particulier les consultations chez des médecins généralistes par les membres des régimes d'assurance maladie.

**Méthodes:**

Il s'agissait d'une étude rétrospective sur les données des régimes d'assurance maladie. La méthode d'imputation médiane a été utilisée pour traiter la prévalence des maladies chroniques manquantes et non déclarées. L'analyse de régression logistique multivariée a été utilisée pour évaluer les effets de la prévalence des maladies chroniques, les tranches d’âge et le nombre de consultations chez des médecins généralistes par an.

**Résultats:**

L’étude montre que la prévalence de l'asthme était significativement associée à plus de trois consultations chez le médecin généraliste (OR = 1.081; 95% CI = 1.008–1.159), de même que la prévalence du diabète de type 2 (OR = 1.087; 95% CI = 1.027–1.152), alors que la prévalence de l'hyperlipidémie (OR = 0.92; 95% CI = 0.875–0.97) était plus susceptible d’être associée à moins de trois consultations chez le médecin généraliste. La prévalence de l'hypertension était associée à plus de trois consultations chez le médecin généraliste par an (OR = 1.132; 95% CI = 1.017–1.26).

**Conclusion:**

Cette étude montre que la taille du régime d'assurance maladie et la prévalence de maladies chroniques telles que l'asthme, le diabète de type 2, l'hyperlipidémie et l'hypertension sont liées aux consultations chez le médecin généraliste. Les médecins généralistes et les programmes de soins utilisés par les régimes d'assurance maladie devraient accorder une attention particulière à certaines maladies présentant un taux de prévalence élevé afin de mieux les prendre en charge.

## Introduction

General practitioners’ (GPs) services have been shown to be a significant determinant of population health, effective cost-containment and promotion of equity objectives.^1^ Unger et al.^2^ showed that GPs were the most common providers of chronic disease primary care, with over 90% of respondents reporting that they had visited a GP at least once in the past 12 months. Utilisation data on GPs’ services by medical scheme members report an average of three annual visits. Barnes, Jonsson and Klim^3^ report that Canadian patients visit doctors more often (4.9 annual GP visits) than Australian patients (2.3–3.6 visits), whilst Harris^4^ contends that on average Australians visit a GP five times per year.

### Key focus

Medical scheme members are entitled to certain benefits that the schemes have to cover in full. These are called Prescribed Minimum Benefits (PMBs), and the PMB Chronic Disease List is a list of conditions which all medical schemes need to cover on all the plans they offer to their members. This cover includes funding for diagnosis, treatment and ongoing care for the listed conditions.^5^ However, from a member's perspective there is still a lack of understanding of what these benefits actually entail. A recent survey by Old Mutual Consulting Actuaries^6^ revealed that 85% of members do not understand their PMB entitlements, or where to access PMBs. However, a greater part of the problem is how these benefits are communicated to members.

With regard to PMBs, schemes develop protocols to manage the use of benefits. Such protocols would specify, for example, types of tests, investigations and number of consultations.^7^ Non-adherence to some of the guidelines can have unintended consequences for the member, such as denial of benefits that a member is entitled to. Some schemes require members to register on disease management programmes prior to entitlement to such benefits. Consequences of not registering on such programmes are outlined in the medical schemes’ rules, which include cases where an unlimited benefit such as a PMB could be considered as a day-to-day benefit, thus unknowingly compromising member's day-to-day benefits, which are limited.

Literature reviews reveal inconsistencies or variation in how protocols or treatment guidelines for the PMB Chronic Disease List are employed, in particular with regard to number of consultations per annum, which is also a proxy for a benefit. In some guidelines patients who suffer from asthma and use chronic medication are entitled to a treatment plan that allows them two visits to a pulmonologist per year; two visits to a GP or physician; and tests such as peak-flow evaluations.^8^ For the purposes of this article we use annual average visits to the GP as a proxy for access to benefits, and find associations with select chronic diseases. This seeks to advance knowledge on GP visits for monitoring and managing of chronic conditions and also as a tool to control costs.

#### Background

Quantifying the impact of chronic disease on healthcare use can assist in estimating the return on investment of health promotion and other policies designed to prevent chronic diseases or better manage the costs associated with them.^9^ Medical schemes employ managed care programmes to monitor utilisation and control costs; these programmes include protocols and guidelines that also prescribe the number of visits to a GP. In The Netherlands a GP is responsible for the primary care of an average of 2350 patients.^10^ Literature reveals that Dutch GPs are the ‘gatekeepers’ of the healthcare system and provide most routine medical care and diagnostic evaluations for their patients. A patient can visit a specialist only after a GP referral.^11^ Other studies have also shown that delivering optimal health care for chronic illnesses requires health systems to move from a reactive approach to a proactive one.^12^

#### Trends

Long-term conditions are chronic illnesses that greatly affect people's lifestyles and require ongoing management over years or decades.^13^ Chronic conditions such as diabetes, heart disease and chronic obstructive pulmonary disease affect over 17.5 million people in the United Kingdom (UK).^14^ Approximately 75% – 85% of healthcare expenditure in the UK is related to chronic disease.^15^ Data show that 60% of people aged over 65 years have a chronic disease, and this is set to double in next 10 years. The literature further illustrate that in the UK 80% of GP consultations and more than half of hospital bed usage relates to a long-term condition.^16, 17, 18^

#### Rationale

In the absence of complete and accurate data, measuring the effect of primary health service use and chronic disease management programmes becomes difficult to assess. Prevalence data are frequently collected through surveys based upon self-reports of disease.^18^ Literature shows that people tend to under-report the presence of chronic disease; under-reporting of HIV and AIDS cases, for instance, is a common problem in HIV epidemiology and often skews epidemiological projections.^19^ Other epidemiological studies have dealt with skewed or missing cases, as has the work of Acuna and Rodriguez.^20^ It is known that missing data can introduce bias into estimates derived from a statistical model.^21^ Missing data and under-reporting of chronic conditions are also key challenges in the medical scheme environment, as reported in the Council for Medical Schemes report.^22^

Another example is HIV reporting in the mining sector. A mining company such as Implats provides treatment programmes for its employees through its own medical facilities and in-house medical scheme; however, employees may choose to receive treatment through external medical facilities which do not report statistics to the company, or through government-provided systems. As a result, HIV and AIDS prevalence levels and other statistics related to the impact of the virus are not known with absolute certainty.^23^ McLeod^24^ further states that the chronic diseases list covers the majority of people with chronic conditions, but warns that this would underestimate the burden of chronic disease in medical schemes.

Enders^25^ reviewed some of the recent methodological advances related to missing data, and provides an overview of two ‘modern’ analytical options: direct maximum likelihood estimation and multiple imputations. In the current article we considered multiple imputations for dealing with missing data.

#### Objectives

The objective of this study was to examine the association between the prevalence of selected chronic diseases on health service use, in particular visits to GPs. The current work seeks to identify specific chronic diseases that may need more attention and can be better managed sooner.

#### Contribution to field

This study investigated factors that are associated with primary healthcare use, in particular GPs’ services. Some of the factors included most prevalent chronic disease associated with visits to a GP. The findings of this study are essential in illustrating the significant role of primary care in managing care for patients, and also identifying chronic diseases that need more attention and monitoring and can be better managed sooner. The study seeks further to enhance understanding of some of the best practice literature in developing and determining clinical guidelines associated with treating and managing chronic diseases. The study uses GP visits as a proxy for accessing chronic benefits.

## Ethical considerations

The current study was not a clinical trial study, and therefore did not directly involve treatment of patients. The data were assessed and only reported at consolidated level for privacy and confidentiality.

## Methods

### Materials

The data used were sourced from the annual statutory return submissions which schemes submit to the Office of the Registrar. The data were captured on the annual statutory returns portal, then exported onto Microsoft Excel spreadsheets prior to the analysis phase.

### Setting

Data analysed included open and restricted schemes that were registered during the assessment period (data observed in 2009). Inclusion criteria were schemes that submitted complete data on the variables of interest.

### Design

This was a retrospective cross-sectional study which included 109 medical schemes that were registered and operational in 2009. A purposive sampling technique was used to select schemes based on specific characteristics: registered schemes for the period under review and completeness of data. The study was representative in terms of the number of schemes, beneficiaries covered and number of benefit options. A sample of schemes represented 99.8% of the private-sector beneficiaries and 99.1% of registered benefit options in 2009.

### Procedure

The total number of visits by beneficiaries of each scheme in each year was extracted from the utilisation section of the annual statutory return data submissions. This was then weighted to account for the number of beneficiaries in each scheme. The average age of beneficiaries was computed at scheme level (Table 1). This was further organised into two strata, namely schemes with average member age of more than 35 years, and those with less than or equal to 35 years. This cut-off was motivated by the findings of a study by Aung, Recehl and Odermatt,^26^ which showed that being younger than 35 years was a main barrier to accessing primary healthcare services. A study by Fuster, Voute, Hunn and Smith^27^ revealed that 41% of all deaths in South Africa were due to heart disease, and this occurred in people 35–64 years of age. Furthermore, actuarial projections in South Africa suggest that chronic diseases are expected to increase, with HIVand AIDS ravaging those aged 18–35 years.^28^ The report further highlights the alarming fact that South Africa is already losing a significant amount of people in the workforce age group of 35–64 years because of cardiovascular disease.^28^

**TABLE 1:** Covariates under investigation: demographic characteristics.

Medical schemes	Average number of visits to a GP per beneficiary per annum
**Scheme type**	
Open scheme	Medical schemes that freely admit everyone
Restricted schemes	Employer group schemes which only admit applicants belonging to a specific employment sector
**Scheme size**	
Large	More than 30 000 beneficiaries
Medium	More than 6000 principal members but not more than 30 000 beneficiaries
Small	All schemes with less that 6000 principal members
**Scheme age strata**	
> 35 vs. ≤ 35	Average age of beneficiaries at scheme level was stratified by > 35 and ≤35 years

Other covariates considered for predicting average number of visits to a GP included a select list of chronic diseases The following 10 selected chronic conditions are those most prevalent with the medical schemes:^23, 29^

- Hypertention
- Hyperlipideamia
- Asthma
- Coronary artery disease
- HIV
- Hypothyroidism
- Epilepsy
- Diabetes mellitus type 1
- Diabetes mellitus type 2
- Cardiac failure.

Chronic disease permeates several aspects of health service utilisation, and can be implicated in many diagnoses; therefore, all services for all relevant ICD-10 diagnostic codes were included. Prevalence of chronic disease was defined by counting every beneficiary who has any of the selected chronic conditions; where beneficiaries had multiple conditions, each condition was counted separately.

### Statistical analysis

Descriptive statistics were calculated to characterise the distribution of chronic disease in the sample population. The median imputation method was employed to deal with missing and unreported cases. This is one of the most frequently used methods, especially when the distribution of values of a given feature is skewed.^30^ According to Durrant,^31^ the imputation method reduces non-response bias due to missing values. The median imputation method consists of replacing the missing data for a given feature (attribute) with the median of all known values of that attribute in the class where an instance is missing. The capping or flooring approach was employed to deal with the outliers.^32^

Multivariate logistic regression analysis methods were employed to assess the effects of the prevalence of chronic illnesses on visits to a GP. Average annual GP visits were used to enhance ability of the statistical models to estimate the variance in utilisation attributable to chronic disease.^33^ The outcome variable was stratified into two groups to form a dichotomous outcome: schemes with average annual visits to a GP > 3 and those with visits ≤ 3.

Continuous measurement of variables such as prevalence of selected conditions such as HIV, asthma, hypertension, diabetes types 1 and 2, epilepsy, hyperlipidaemia, coronary artery disease, hypothyroidism and cardiac failure were included as covariates in the multivariate logistic regression model. The average age of beneficiaries in schemes, scheme type, and scheme size were also considered as covariates in the model. We conducted all the analysis using SAS software, version 9.2 (SAS Institute Inc., Cary, NC). Statistical significance tests were conducted at α = 0.05 level (*p* < 0.05); odds ratio (OR) and the 95% confidence intervals (CIs) were also reported.

## Results

### Participant characteristics

The sample of schemes analysed represented 99.8% of the private-sector beneficiaries and 99.1% of registered benefit options for the 2009 data. The median number of visits to a GP in 2009 was 3.2 (IQR = 2.4–3.7), and the median of the average age of beneficiaries was 32.91 years (IQR = 30.1–36.6) (Table 2). The median prevalence rate per 1000 beneficiaries for hypertension was 109.5 (IQR = 82.8–159.0), followed by hyperlipidaemia at 52.9 (IQR = 29.9–78.9). The median prevalence rate for asthma was 27.9 (IQR = 20.3–37.5), hypothyroidism 19.8 (IQR = 11.7–31.5) and cardiac failure 4.2 (IQR = 1.4–7.1) per 1000 beneficiaries. The prevalence of beneficiaries with type 2 diabetes mellitus was 30.1 (IQR = 20.7–38.3) per 1000 beneficiaries. The prevalence of select chronic diseases per 1000 beneficiaries for the medical schemes considered in the current study. The average number of GP visits for restricted schemes was slightly higher than in open schemes (3.3 compared to 2.9 visits [Table 2]).

**TABLE 2:** Prevalence of select chronic diseases per 1000 beneficiaries by scheme type.

Variables	Total (*N* = 109)	Open (*N* = 33)	Restricted (*N* = 76)	Median	IQR	*p*-value
GP visits per annum	3	2.9	3.3	3.2	2.4-3.7	0.065
Asthma	26.7	28.9	23.5	27.9	20.3-37.5	0.09
Cardiac failure	6	5	7.4	4.2	1.4-7.1	0.332
Coronary artery disease	14.9	16.9	11.8	14.6	7.7-23.3	0.958
Type 1 diabetes	6.5	7.3	5.2	4.4	2.8-7.9	0.774
Type 2 diabetes	28.5	29.2	27.5	30.1	20.7-38.3	0.966
Epilepsy	7.1	7.8	6	7.2	5.2-9.7	0.63
HIV	7	6.4	7.8	6	0.2-12.9	0.719
Hyperlipidaemia	48.4	52.8	42	52.9	29.9-78.9	0.222
Hypertension	107.7	113.3	99.3	109.5	82.8-159.0	0.887
Hypothyroidism	18	19.1	16.2	19.8	11.7-31.5	0.34
**Benefits paid per beneficiary per month (ZAR)**						
GPs	58.7	52.7	67.6	59.8	44.7-70.7	0.0002*
Total hospitals	293	315.9	258.9	313.2	251.5-392.2	< 0.0001*
Total benefits	790.1	827.3	734.8	860.2	707.8-1071.4	< 0.0001*

IQR, interquartile ranges.

* *p* < 0.05; 1 ZAR/$ = 8.8

The prevalence of chronic disease in open schemes was slightly higher than in restricted schemes, except for HIV cases (7.8 compared to 6.4/1000 beneficiaries) and cardiac failure (7.4 compared to 5.0/1000 beneficiaries). The difference in average expenditure on GP visits between open and restricted schemes was not significant, at R52.70 compared R67.60 per beneficiary per month. Overall, total benefits paid to providers were higher in open schemes than in restricted schemes.

Results revealed that the prevalence rate of cardiac failure in the older profiled schemes was nearly twice that in the older group (Table 3). The prevalence of coronary artery disease in the older profiled schemes was nearly three times that in the younger profiled schemes. Prevalence rates for hyperlipidaemia, hypertension, hypothyroidism and type 2 diabetes were twice as high for the older profiled schemes as for the younger profiled schemes. Average expenditure on GPs was not significantly different between the younger and the older profiled schemes, at R58.70 compared to R49.52 per beneficiary per month.

**TABLE 3:** Age stratum of beneficiaries in schemes.

Beneficiaries	<35 years (*N* = 39)	≥ 35 years (*N* = 70)	*p*-value
Average number of GP visits per year	3.2	2.5	0.6
**Prevalence of select chronic diseases per 1000 beneficiaries**			
Asthma	25.7	31.1	0.0*
Cardiac failure	5.3	8.6	<0.0001*
Coronary artery disease	11.3	30.1	0.0*
Type 1 diabetes	6.3	7.4	0.1
Type 2 diabetes	25.2	42.8	<0.0001*
Epilepsy	6.4	9.7	<0.0001*
HIV	7.2	6.1	0.3
Hyperlipidaemia	38.4	91.3	<0.0001*
Hypertension	89.9	184	<0.0001*
Hypothyroidism	13.7	36.1	<0.0001*
**Benefits paid per beneficiary per month (ZAR)**			
GPs	58.7	49.5	0.3
Total hospitals	293	397.3	<0.0001*
Total benefits	790.1	995.8	<0.0001*

* *p* < 0.05; 1 ZAR/$ = 8.8

**FIGURE 1:**
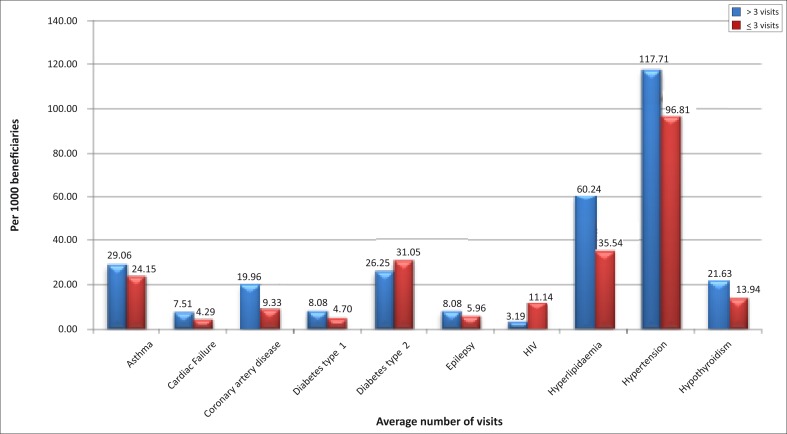
Prevalence rates of selected chronic diseases by General Practitioner visit stratum at scheme level.

Hypertension was the most prevalent chronic disease, with 117.17 compared to 96.81 per 1000 beneficiaries for the stratum, and an average of 3+ visits compared to ≤3 visits for the stratum (Figure 1). The second most prevalent was hyperlipidaemia with 60.24 compared to 35.54 per 1000 beneficiaries and 3+ visits compared to ≤3 visits. Asthma and diabetes type 2 were the third and fourth most prevalent chronic diseases in the data presented.

**FIGURE 2:**
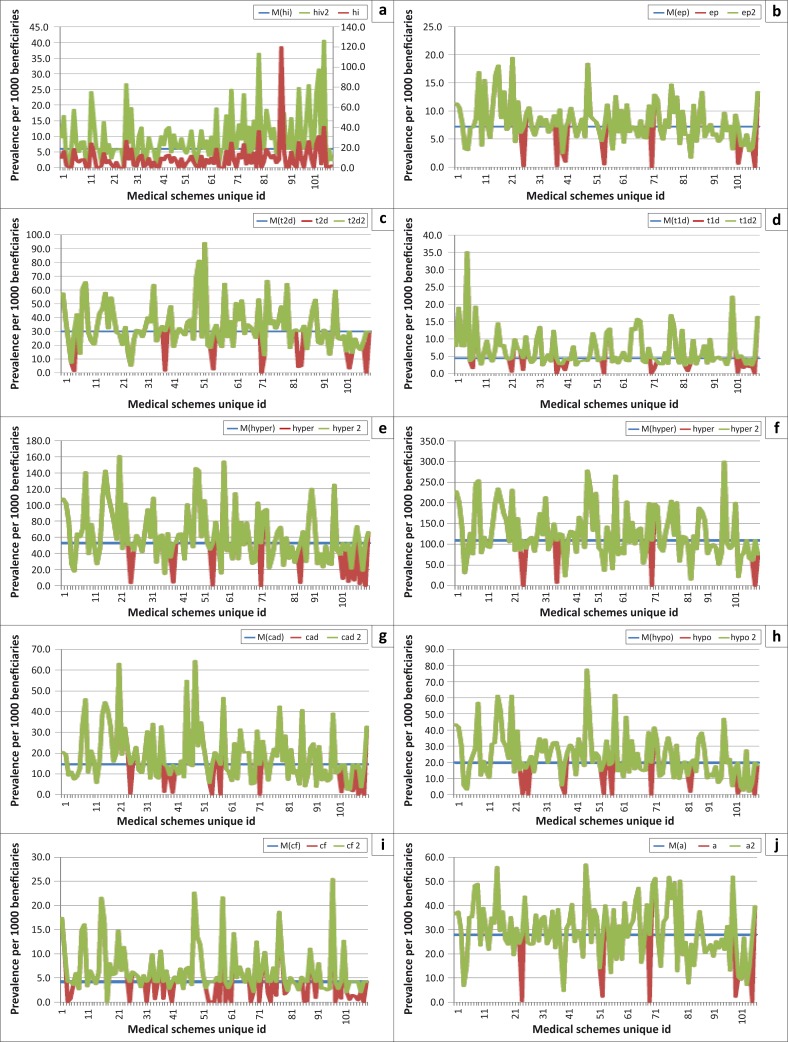
Graphic representation of the selected chronic diseases (median vs. reported vs. adjusted prevalence rates).

Missing and non-reported cases were identified and cross-validated by a comparison analysis of the conditions, looking at previous years’ data on the same schemes and also triangulating with the Risk Equalisation Fund data submissions. Risk-equalisation is a mechanism that was proposed for achieving equity and efficiency in regulated private health insurance markets. (The Risk Equalisation Fund has been operating in shadow mode since 2005, with data being collected from schemes but no money changing hands; it was scheduled to be implemented 2012–2013.) All of the adjusted cases were denoted with the suffix ‘2’, and these were compared to the reported data. The median in each plot was denoted with the prefix ‘M’ (Figure 2).

A measurable deviation between reported cases and adjusted cases was noted. The most prevalent non-responses were cases of HIV, cardiac failure and hypothyroidism. In the first model, denoted by ML1, we employed a rule of thumb where all non-reported cases of less than five were replaced by the median. The second model, denoted ML2, is where all reported cases less than the 50th percentile for chronic prevalence were replaced by the median. In the third and last model, denoted ML3, all reported cases smaller than the 10th percentile were replaced by the median. Capping for all three models was at the 90th percentile. Comparative statistics on the results of the three fitted models are discussed in the next section.

### Modelling prevalence of chronic diseases associated with primary healthcare visits

All three criteria for assessing goodness of fit suggested that ML3 was a better fit for modelling GP visits, and the test statistics confirming this are Chi-square = 26.14, *p* = 0.0249 (Table 4). Results obtained from fitting this model are presented (Table 5). Regression results for ML3 revealed that scheme size, asthma, type 2 diabetes, hyperlipidaemia and hypertension were significantly associated with GP visits.

**TABLE 4:** Summary of fitting predictors of primary health care use.

Criterion	ML1	ML2	ML3
-2 Llog likelihood	106.366	111.401	99.39
AIC (smaller is better)	136.366	141.401	129.39
SC (smaller is better)	176.736	181.771	169.76

AIC, Akaike Information Criterion; SC, Schwarz Criterion are criterion for the measure of the relative goodness of fit of a statistical model; ML1, Mixed Linear Model 1; ML2, Mixed Linear Model 2; ML3, Mixed Linear Model 3.

**TABLE 5:** Multivariate logistic regression results for prediction.

Covariates	OR	95% LCI	95% UCI	*p* - value
Scheme type (restricted vs. open)	2.758	0.759	10.02	0.1232
Age stratum (> 35 vs. <35)	2.656	0.563	12.54	0.2174
Scheme size (large vs. small)	0.333	0.093	1.19	0.0907*
Scheme size (medium vs. small)	0.161	0.039	0.661	0.0343**
Asthma	1.081	1.008	1.159	0.0291**
Cardiac failure	1.108	0.893	1.375	0.3522
Coronary artery disease	0.947	0.862	1.04	0.2537
Type 1 diabetes	0.941	0.849	1.044	0.2543
Type 2 diabetes	1.087	1.027	1.152	0.0041**
Epilepsy	1.199	0.875	1.644	0.2597
HIV	1.049	0.967	1.138	0.252
Hyperlipidaemia	0.92	0.87	0.973	0.0037**
Hypertension	1.132	1.017	1.26	0.0233**
Hypothyroidism	0.997	0.975	1.019	0.7605

* *p* < 0.1

** *p* < 0.05, OR, odds ratio; LCI, lower confidence interval; UCI, upper confidence interval; ≤3 visits versus 3+ visits.

Results indicate that average age of beneficiaries at scheme level, scheme type, and prevalence of cardiac failure, coronary artery disease, type 1 diabetes, epilepsy, HIV and hyperlipidaemia are not significant in terms of average number of GP visits (Table 5). They further illustrated that those on small schemes were likely to have more than three visits compared to those on medium schemes (OR = 0.16; 95% CI = 0.039–0.661); otherwise there were no significant differences between small and large schemes.

The data further showed that prevalence of asthma was significantly associated with GP visits (OR = 1.081; 95% CI = 1.008–1.159). Thus, asthma prevalence rates were likely to be associated with more than three GP visits, similar to the prevalence of type 2 diabetes, with OR = 1.087; 95% CI = 1.027–1.152. The prevalence of hyperlipidaemia (OR = 0.92; 95% CI = 0.875–1.644) was more likely to be associated with less than three GP visits. Lastly, prevalence rates for hypertension were likely to be associated with more than three GP visits per year (OR = 1.132; 95% CI = 1.017–1.26).

## Discussion

Chronic illnesses greatly impact on the patients’ way of life and require ongoing monitoring and management. Proactively managing such illnesses through educational and continuous monitoring methods could certainly improve the health status of the country. A survey by Seghieri et al.^34^ confirmed that informing patients about their care and how to manage condition-related symptoms may lead to more effective chronic disease management and improved health status. Informing patients about their care should incorporate effectively communicating to patients or beneficiaries as to their benefit entitlement, particularly PMBs.

*The Medical Schemes Act*^35^ requires that limitation on disease coverage be developed on the basis of evidence-based medicine. For instance, some schemes specify in their rules that patients who suffer from asthma and use chronic medication are entitled to a treatment plan that allows them two visits to a pulmonologist per year; two visits to a GP or physician; and tests such as peak-flow evaluations. All these data should be communicated to the member, as should the implication of not registering on a scheme's chronic disease management programme as per scheme rules, and how this could affect their day-to-day benefits.

The average number of GP visits in the private medical schemes data in 2009 was three.^36^ This is slightly lower than the Canadian average (Canadian patients visit doctors more often than Australian patients, making 4.9 GP visits annually compared to 2.3–3.6 in Australia^3^); however, Harris^4^ states that on average Australians visit a GP five times per year.

According to an HLC Financial Services publication, one of the biggest open schemes in South Africa covers four GP consultations per year for each approved chronic disease.^37^ Our study showed that the prevalence of asthma was significantly associated with more than the average of three annual visits to a GP (OR = 1.081; 95% CI = 1.008–1.159; *p* = 0.0291). These results are consistent with the data analysed by Barnes^38^, where it was recommended that patients with mild asthma required three to five visits to their GP annually. Their study further illustrated that individuals with moderate asthma appeared to contribute more to the burden of asthma care than those with severe asthma.

Our study also revealed a significant association between prevalence of type 2 diabetes and the average number of GP visits per year (OR = 1.087; 95% CI = 1.027–1.152, *p* = 0.0041). Thus, beneficiaries with type 2 diabetes were likely to make more than three visits to a GP. These results are consistent with the literature; for instance, a study by Johnson, Rabi, Edwards and Balko^39^ showed that adults with diabetes made more than nine GP visits on average, whilst those with no diabetes made just over five. Another study by Bottomley and the T2ARDIS Steering Committee^40^ showed that patients with type 2 diabetes visited their GP on average five times a year, and the GP visited them at home once every two years. Rutten, Van Eijk, De Nobel, Beek and Van der Helden^41^ studied the relationship between the number of clinic visits for diabetes patients and changes in blood glucose control; their study illustrated that at the frequency of two visits per year, HbAl decreased in 31% of patients, with three or four visits in 35%, and with five or more in 79% of patients (*p* < 0.005).

Our study also revealed a significant association between hyperlipidaemia and primary healthcare use. This is consistent with the literature; Eaton et al.^42^ state that family physicians have potential to make a major impact on reducing the burden of cardiovascular disease through the optimal assessment and management of hyperlipidemia. Their study also found that the frequency of primary care visits seemed to be fairly uniform for both well-controlled (average 2.2 visits per year) and uncontrolled hyperlipidaemic (4.2 visits per year) patients. Lastly, our study revealed a significant association between hypertension and GP visits (OR = 1.132; 95% CI = 1.017–1.26).

## Limitations of the study

One of the limitations of the study is that risk factors associated with chronic diseases were not explored. These include tobacco use, obesity or diet, hypercholesterolaemia, alcohol abuse, sedentary lifestyle and certain infectious diseases.^43^ Another limitation is that we did not risk-adjust the reported chronic prevalence for particular age groups, genders and ethnic groups. Al-Windi^44^ has shown that a higher proportion of females than males had one to five or more than five GP consultations per year. According to Polisson,^45^ demand for GP visits is most likely driven by health status and, for women, childbirth.

It is also known that some chronic diseases are more prevalent in certain age groups and genders; hypothyroidism, for example, is more common in older persons, especially women, principally due to the rising incidence and prevalence of auto-immune thyroiditis.^46^ A study by Pillar, Levy, Holcberg and Sheiner^47^ showed that treated hyperthyroidism was not associated with adverse perinatal outcome; however, hyperthyroidism was found to be an independent risk factor for caesarean delivery. Hyperthyroidism is common, affecting approximately 2% of women and 0.2% of men.^48^ This further emphasises the importance of risk factors and risk adjustments to get a more holistic and better perspective of the results.

Lastly, data was analyses were at scheme level; a wide-ranging assessment of chronic diseases and primary healthcare benefits at benefit option level could certainly enhance the findings of the current study. However, it was illustrated during the Risk Equalisation Fund shadow period that even though benefit options differ in design, the CDL is about the same in each option.^24^

## Recommendations

Recommendations arising from the current study are that primary healthcare services have an essential role in the private health sector, in particular in managing chronic disease. The results obtained and this study adds value to managed care interventions employed by schemes in advocating more awareness, educating members and continuous monitoring of chronic diseases. This proactive approach is vital for avoiding hospitalisations.

Other factors were not taken into account in this study, such as risk factors and risk adjustments; however, it is recommended that patients with chronic conditions visit their GP frequently to identify specific problems that need more attention and can be better managed sooner. Some of the select chronic diseases need more attention than others; also, the severity of the condition impacts on number of visits to a GP. All these considerations should be taken into account when designing protocols and guidelines for provision of benefits. Furthermore, there is a need to review protocols employed by the schemes for provision of PMBs, to ensure that these are consistent with recent best practice and comply with the *Medical Schemes Act*, in particular Regulation 15.

Schemes need to educate members on their benefit entitlement, in particular chronic benefits, and also on the consequences of not registering on chronic diseases programmes. Protocols and guidelines used as clinical risk measurement tools should be communicated to members; these should also outline the minimum standards required to control or manage the conditions. Such protocols and clinical risk measures should not compromise the health status of beneficiaries for cost-effectiveness.

## Conclusion

The current study employed MI to account for missing data and outliers. This method allowed for a more complete set of data, to enhance the results of the multiple regression analysis model. Using these statistical methods to deal with the shortcomings of the data from medical schemes, we showed that scheme size, asthma, type 2 diabetes, hyperlipidaemia and hypertension were related to the annual number of GP visits. Some of the key chronic diseases considered in the current study were found not to have a significant link with number of GP visits, an indication that estimating the effect of chronic disease on health service use is complex.

These results illustrate the minimum number of visits required to manage select chronic diseases. The findings of the current study further enhance the role of primary health care and preventative measures employed by managed care entities as an effective tool to effectively avoid costly hospitalisation.
